# Early Intracellular Trafficking and Subsequent Activity of Programmed Cell Death in Channel Catfish Macrophages Infected with *Edwardsiella ictaluri*

**DOI:** 10.3390/microorganisms8111649

**Published:** 2020-10-24

**Authors:** Lidiya P. Dubytska, Ronald L. Thune

**Affiliations:** 1 Department of Pathobiological Sciences, Louisiana State University School of Veterinary Medicine, Baton Rouge, LA 70803, USA; lidiya_dubytska@subr.edu; 2 School of Animal Science, Louisiana State University Agricultural Center, Baton Rouge, LA 70803, USA

**Keywords:** *Edwardsiella ictaluri*, type-3 secretion system, Edwardsiella-containing-vacuoles, autophagy, apoptosis

## Abstract

The development of *Edwardsiella*-containing-vacuoles (ECV) and the ability of *Edwardsiella ictaluri* to survive and replicate within macrophages suggests a unique process relative to normal phagosomal/lysosomal maturation and programed cell death. Developing ECV showed that endosomal membrane markers Rab5, EEA1, and Rab7 were all detected in both the wild type (WT) and an *E. ictaluri* type-3 secretion system (T3SS) mutant, 65ST. Co-localization with Lamp1, however, was significantly lower in the WT. The host cell endoplasmic reticulum marker, calnexin, co-localized to 65ST ECV significantly more than WT ECV, while Golgi vesicle marker, giantin, was recruited to WT ECV significantly more than 65ST. The autophagosomal marker LC3 was significantly lower in WT than in 65ST and Western blotting demonstrated significantly greater induction of the membrane localized, lipidated form, LC3-II, in 65ST ECV than in WT ECV. Activity of the apoptosis initiator caspase-8 increased post-infection in 65ST and was significantly lower in WT-infected cells. Executioner caspase-3/7 activity also increased significantly in 65ST-infected cells compared to WT-infected cells. Repression of apoptosis was further demonstrated with flow cytometry using Alexa Fluor 647-labeled Annexin V and propidium iodide. Results indicate that WT ECV fused with early and late endosomes but that phagosomal/lysosomal fusion did not occur. Additionally, WT-infected cells recruited Golgi vesicles for vacuolar size increase and bacterial growth material, and both autophagy and apoptosis were repressed in the WT. This activity was all based on the function of the *E. ictaluri* T3SS.

## 1. Introduction

The Gram-negative bacterial pathogen *Edwardsiella ictaluri* causes enteric septicemia of catfish (ESC), an economically significant disease of farmed-raised channel catfish, *Ictalurus punctatus*. Previous work demonstrated survival and replication of *E. ictaluri* in channel catfish head-kidney-derived macrophages (HKDM) [1] and also identified a type-3 secretion system (T3SS) that is essential for *E. ictaluri* virulence and intracellular replication [2]. A total of nine T3SS effector proteins have been identified in the *E. ictaluri* genome to date [3], one on each of the two native *E. ictaluri* plasmids [4] and seven encoded on the chromosome. Each of these proteins was shown to be actively translocated from the bacterial cell, directly through the bacterial outer envelope and membrane of the *Edwardsiella*-containing-vacuoles (ECV), to the host cell cytoplasm by the T3SS [3]. Combined with data on regulation of the T3SS [5], on pH modulation of the ECV [6], and on the activity of an *E. ictaluri* acid-activated urease enzyme [1], development of a model for the early establishment of an *E. ictaluri* infection in HKDM and development of the ECV is possible. Briefly, once *E. ictaluri* enters the HKDM, the ECV are immediately acidified by the vacuolar ATPases of the host cell [6]. This results in upregulation of expression of the T3SS by the two-component regulatory proteins EsrA and EsrB [5], as well as activation of the *E. ictaluri* acid-activated urease [1]. The urease enzyme utilizes urea produced by the host cell arginase enzyme to produce ammonia, which acts to neutralize the acidic environment of the ECV and increase the pH to a level that is conducive to *E. ictaluri* replication (>pH 5.0) [1,6]. Neutralization of the ECV also triggers translocation of the *E. ictaluri* T3SS effectors across the membrane of the ECV into the host cell cytosol [3].

The development of the ECV and the ability of *E. ictaluri* to survive and replicate within HKDM suggests a unique process relative to normal phagosomal/lysosomal maturation pathways and resistance to the normal pathways of programed cell death. Although the early model of infection described above gives insight into the beginning of the disease process, the action of the T3SS and its associated effectors defines the actual disease symptoms and pathology that occurs. A functioning T3SS in a pathogen that survives and replicates in an intact phagocytic vacuole suggests that the T3SS has manipulated the host cell physiology and altered the development of the vacuole. In addition, failure of the host to kill the wild-type *E. ictaluri* (WT) suggests that host functions of programmed cell death are neutralized by the bacterium. Consequently, studies were initiated to describe the development of the ECV through detection of indicator protein markers in the ECV membranes and to explore possible mechanisms that mediate intracellular survival in light of programmed cell death in order to further develop the model for *E. ictaluri* pathogenesis.

## 2. Materials and Methods

### 2.1. Bacterial Strains

*Edwardsiella ictaluri* WT strain 93–146 and a T3SS knockout mutant 65ST [2] were grown for 16–18 h at 28 °C to an OD_600_ = 1.8 to 2.0 in porcine brain heart infusion (BHI) broth. All strains grown in broth were aerated on a Cel-Gro tissue culture rotator (Lab-Line, Inc., Melrose Park, IL, USA).

### 2.2. Antibodies, Probes and Reagents

Rabbit polyclonal antibody against early endosomal antigen 1, EEA1, the small GTPase, Rab5, and the endoplasmic reticulum marker, calnexin, were purchased from GenScript (Piscataway, NJ, USA, Catalogue # A01514, A01302, and A01192, respectively). Rabbit polyclonal antibody against late endosomal marker Rab7 was purchased from Osenses (Keswick, SA, Australia # OSR00306W). Rabbit polyclonal antibody against Lamp-1 and giantin were obtained from Abcam (Cambridge, MA, # AB24170 and AB24586). Rabbit polyclonal antibody against autophagy marker LC3/LC3-II was purchased from Thermo Scientific (Waltham, MA, USA, # PA1-16931). Bacteria were revealed by immunofluorescence using mouse monoclonal antibody Ed9 against *E. ictaluri* lipopolysaccharide [2,7,8] or Oregon Green 514 succinimidyl ester (Invitrogen, Carlsbad, CA, USA). The secondary antibodies used were Alexa Fluor 555 or 594 goat anti-rabbit and fluoresceine goat anti-mouse (Molecular Probes). Nuclei were stained with ‘6-diamidino-2-phenylindole, dihydrochloride (DAPI) (Molecular Probes, Eugene, OR, USA).

### 2.3. Infection Procedure

Isolation of the HKDM was as previously described [8]. Cells were diluted to give a final concentration of 1 × 10^7^ cells/mL in channel catfish macrophage medium (CCMM) consisting of RPMI 1640 medium without phenol red or L-glutamine (Lonza, Walkersville, MD, USA) but containing 1X Glutamax (Gibco, Invitrogen Corp., Carlsbad, CA, USA), 15 mM HEPES buffer, 0.18% sodium bicarbonate, 0.05 mM 2-beta-mercaptoethanol (Sigma Chemical Co., St. Louis, MO, USA), and 5% heat-inactivated, pooled channel catfish serum. Both CCMM and RPMI were diluted to the catfish tonicity of 240 mosmol/kg H_2_O by adding sterile deionized/distilled water at a 1:9 ratio (ctCCMM and ctRPMI). One milliliter of the cell suspension was added to each well of a 24-well tissue culture plate containing Biocoat poly-D-lysine coated cover slips (Corning, Bedford, MA, USA) and allowed to adhere for 16 h at 28 °C under 5% CO_2_. Wells were then washed three times with ctRPMI to remove non-adherent cells and covered with 1 mL of fresh ctCCMM. For *E. ictaluri* uptake, bacteria were opsonized for 30 min in normal autologous serum and added to duplicate wells with HKDM cultures at a multiplicity of infection (MOI) of 1 bacterium to 1 HKDM. After infection, plates were centrifuged at 500× *g* for 5 min to synchronize contact of the bacteria with the adhered cell layer and allowed to incubate for 30 min. The medium was then removed, and each well was washed with ctRPMI, after which ctCCMM with 100 μg/mL gentamicin was added for 1 h at 28 °C to kill any remaining extracellular bacteria. Finally, wells were washed once with ctRPMI, after which ctCCMM containing a 1 μg/mL bacteriostatic dose of gentamicin was used to control the extracellular growth of any bacteria released from the cells during the experiment.

### 2.4. Immunofluorescent Microscopy

Infected macrophages were washed with phosphate buffered saline (PBS) and fixed with 4% paraformaldehyde for 20 min at various times after the initial addition of the bacteria. Cells were then washed three times with PBS, permeabilized for 15 min with 0.01% Triton 100, and blocked for 1 h in PBS with 5% goat serum. Coverslips were incubated with primary antibodies directed against Rab5, EEA1, Rab7, LAMP1, calnexin, and giantin for 16 h at 4 C in PBS with 1% goat serum. Bacteria were detected using the primary monoclonal antibody Ed9 [9]. After washing three times in PBS with 1% goat serum, coverslips were incubated for 1 h with secondary antibodies in PBS with 5% goat serum. During this time, macrophage nucleic acids were stained with DAPI. After washing three times again with PBS and once with deionized water, SlowFade Gold antifade (Molecular Probes, Eugene, OR, USA) was added to the cover slips, which were mounted onto glass slides, and cells were observed on a Zeiss Observer Z1 fluorescent microscope (Zeiss, Thornwood, NY, USA). To determine the percentage of bacteria that co-localized with the different intracellular markers, a minimum of 100 ECV were counted for each marker. All assays were performed in triplicate.

### 2.5. Immunoblotting of LC3/LC3-II

Lysates of infected HKDM were separated by SDS-PAGE on 12% polyacrylamide gels and transferred onto PVDF membranes by using an iBlot dry blotting system (Invitrogen) according to the manufacturer’s instructions. Membranes were blocked with 5% skim milk in Tris-buffered saline with 0.1% Tween 20 (Sigma-Aldrich, St. Louis, MO, USA) for 1 h. LC3/LC3-II protein was detected with LC3A/B rabbit polyclonal antibody (Cell Signaling Technology, Danvers, MA, USA). As a loading control, tubulin was detected using rabbit α/β-tubulin antibody (Cell Signaling Technology). Goat anti-rabbit IRDye 800 CW (Li-COR, Lincoln, NE, USA) was used as a secondary antibody, followed by detection on a Li-Cor Odyssey Clx imager.

### 2.6. Caspase Activity

Cultures of HKDM infected with either wild-type *E. ictaluri* or the 65ST T3SS mutant were inoculated into nine replicate wells of a 24-well plate at an MOI of 10 bacteria to one cell. Staurosporine-treated HKDM (1 μM) were used as a positive control. Cells in three wells of each treatment were lysed at 1, 3, and 5 h following addition of the static dose of gentamicin and the staurosporine. Caspase-8 and caspase-9 activity were measured using the Caspase-8/Caspase-9 Apoptosis Assay Kit (Cell Meter, Sunnyvale, CA, USA). Caspase-3/7 activity was measured using the Apo-ONE Homogeneous Caspase-3/7 Assay (Promega, Madison, WI, USA). Fluorescence was measured on a SpectraMax m2 microplate reader (Molecular Devices, Sunnyvale, CA, USA). Caspase activity was expressed as the ratio of infected to uninfected control cells [10].

### 2.7. Assessment of Apoptosis by Flow Cytometry

Apoptosis was detected in HKDM infected with WT and 65ST strains of *E. ictaluri* using Alexa Fluor647-labeled Annexin V (AnnV) and propidium iodide (PI) (ThermoFisher, Waltham, MA, USA) according to the manufacturer’s instructions. Uninfected HKDM and HKDM treated with 1 μM staurosporine were used as controls. After 5 h of infection, macrophages were washed with PBS and removed from the 6-well plate in 100 μL of AnnV binding buffer. The cell suspension was then incubated with 5 μL AnnV and 5 μL of PI in the dark for 15 min at room temperature. Finally, 400 μL of binding buffer was added and the samples were analyzed within 1 h on a BD FACSCalibur cell analyzer (Bectin, Dickinson and Company, Franklin Lakes, NJ, USA). A total of 50,000 cells were counted in each of three replicated experiments. Early apoptotic cells bind Annexin V, a Ca^2+^-dependent phospholipid-binding protein with a high affinity for externalized phosphatidylserine. Propidium iodide stains double-stranded DNA in cells with damaged cell membranes but fails to penetrate and stain cells with intact membranes. Samples were gated on the basis of forward versus side scatter for size, and the results are presented as the percentage of cells that were viable (AnnV−, PI−), early apoptotic (AnnV+, PI−), or late apoptotic/necrotic cells (AnnV+, PI+).

### 2.8. Cytotoxicity Assay

Using HKDM infected with bacterial cultures at an MOI of 10 bacteria to one HKDM, leakage of lactate dehydrogenase (LDH) from the cell cytoplasm was quantified using the CytoTox-ONE Homogeneous Cytotoxicity assay (Promega) at three and five hours post-infection. A spontaneous release control consisted of supernatant from non-infected HKDM. The maximum release control was supernatant from cells lysed with 1% Triton X-100 for one minute. A 100 μL aliquot of each sample was dispensed in triplicate wells of a 96-well plate and LDH activity was determined as per the manufacturer’s protocol. Percentage cytotoxicity was calculated using the following formula: 100% X ((experimental release—spontaneous release)/(maximum release—spontaneous release)), as per the manufacturer’s protocol.

### 2.9. Statistical Analysis

Statistical significance of differences over time for co-localization of *E. ictaluri* WT or 65ST vacuoles carrying Rab5, EEA1, Rab7, Lamp1, calnexin, giantin, or LC3 was determined using a one-tailed unpaired T-test for each time period. Differences for LC3-II, Caspases 3/7, 8, and 9 were analyzed using analysis of variance with Tukey’s post-test to compare the mean of each treatment with every other treatment. The AnnV/PI stained cells were analyzed by analysis of variance with the Bonferroni post-test to compare uninfected cells and 65ST-infected cells to WT-infected cells. The cytotoxicity data were analyzed using analysis of variance and Tukey’s post-test to compare all treatments to each other.

## 3. Results

### 3.1. Immunofluorescence of Vacuolar Membrane Markers

**Figure 1:**
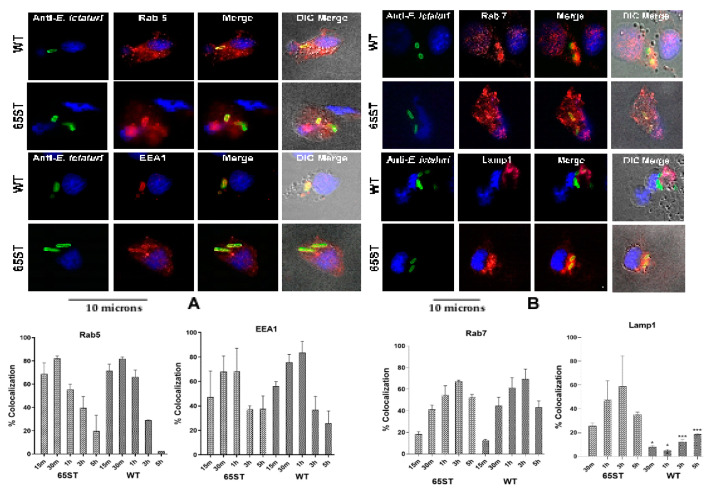
Immunofluorescence micrographs showing subcellular co-localization of early and late endosomal markers. WT and 65ST strains of *E. ictaluri* stain green. The host cell nucleus stains blue. The host cell is visible in DIC in the merged image. The graphs show the percentage of the ECV that col-localized with the given marker over time. (**A**) The early endosomal markers Rab 5 and EEA1 stain red. (**B**) The late endosomal markers Rab7 and Lamp1 also stain red. Results show that %-co-localization of the T3SS mutant 65ST and WT were not significantly different for the early endosomal markers Rab5 and EEA1, or for the late endosomal marker Rab7. Significantly lower levels of Lamp1 in the WT, however, indicate that phagosomal/lysosomal fusion did not occur for the WT. Statistical significances over time were determined using one-tailed unpaired T-test for each period. Data presented as the mean of three sets of 100 cells. Asterisks indicate significant differences of the WT compared to the TT3S mutant 65 ST (* = *p* ≤ 0.01, *** = *p* ≤ 0.001).

Examination for specific vacuolar markers on the developing ECV showed that the percentage of Rab5 positive vacuoles was 58 to 81% in the first hour post-infection for both the wild type (WT) and the T3SS mutant (65ST) but declined to 5–20% at 5 h (Figure 1A). Similarly, there was no significant difference between the WT and 65ST for EEA1, which was from 50 to 80% positive in the first hour and declined to 20–40% at 5 h (Figure 1A). These data indicate that early ECV containing either WT or 65ST both interact with early endosomes. In addition, co-localization of the late endosomal marker Rab7 was not significantly different between 65ST and the WT (Figure 1B). These results for Rab5, EEA1, and Rab 7 indicate that early development of the ECV is similar for both WT and the 65ST T3SS mutant and that both early and late endosomes fuse with the ECV. Co-localization of Lamp1, however, was significantly lower in the WT compared to 65ST Figure 1B). 

**Figure 2:**
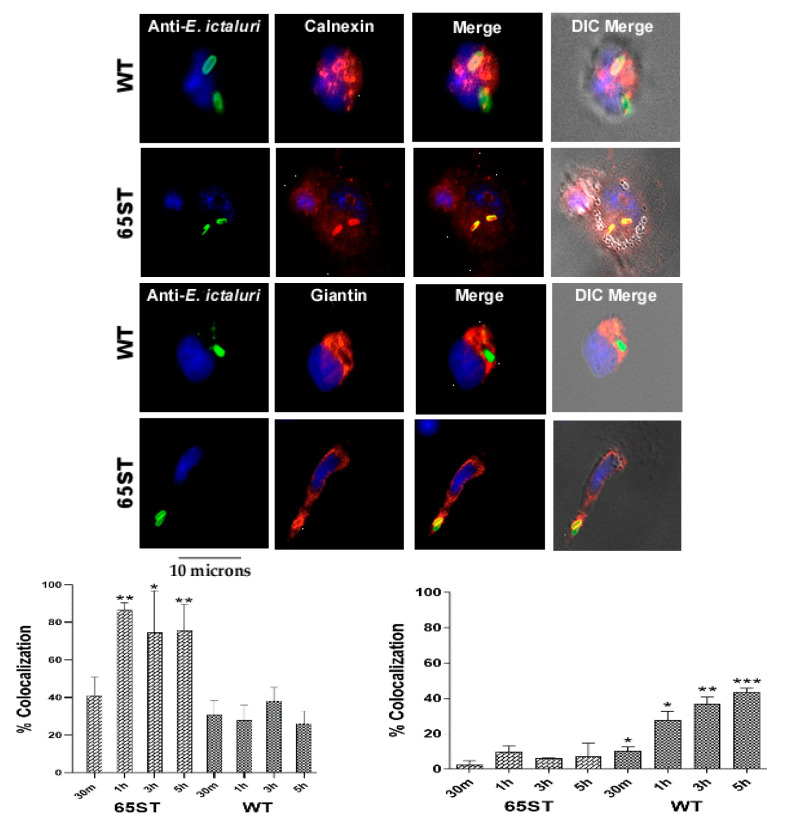
Immunofluorescence micrographs showing that co-localization of the endoplasmic reticulum marker calnexin (red) was significantly greater for ECV in 65ST from 1 to 5 h post-infection than for the WT, while co-localization of the Golgi marker giantin (red) was significantly greater for the WT ECV than for 65ST from 30 min to 5 h PI. The WT and 65 ST strains of *E. ictaluri* stain green in the ECV and the host cell nucleus stains blue. The host cell is visible in DIC in the merged image. The graphs show the percentage of the ECVs that co-localized with the given marker over time. Statistical significances over time were determined using one-tailed unpaired T-tests for each time period. Data presented as the mean of three sets of 100 cells counted. Asterisks indicate significant different differences of the WT compared to the T3SS mutant 65ST (* = *p* ≤ 0.1, ** = *p* ≤ 0.01, *** = *p* ≤ 0.001).

Further analysis showed that the host cell endoplasmic reticulum (ER) marker calnexin co-localized to the ECV of 65ST at a rate of 40% after 30 min and increased to around 60% at 1, 3, and 5 h (Figure 2). Calnexin co-localized to the WT vacuole at a rate of only 25% after 30 min, did not significantly change over time (Figure 2), and was significantly lower than 65ST. At the same time, the Golgi vesicle marker giantin was recruited to 10% of the WT ECV after 30 min and increased significantly to 23%, 35%, and 42% at 1, 3, and 5 h post-infection (Figure 2). Giantin co-localization was significantly greater for the WT than for 65ST, which stayed below 10% at all time points.

**Figure 3:**
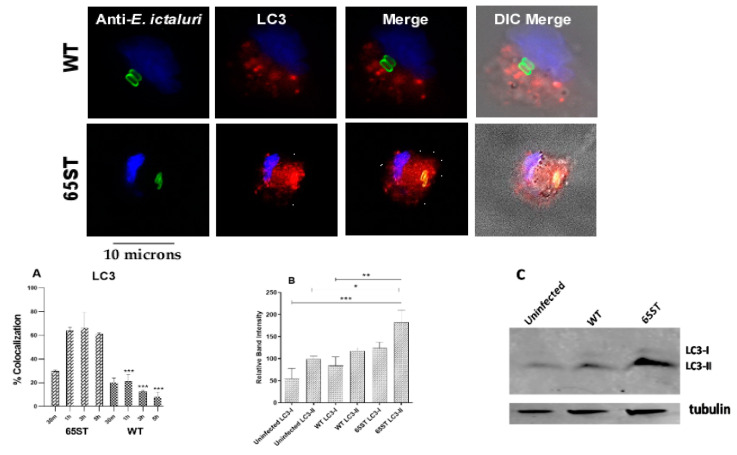
Immunofluorescent micrograph showing the subcellular localization of WT and 65ST mutant of *E. ictaluri* (green) in relation to the autophagy marker LC3 (red) 3 h post-infection. The host cell nucleus is blue and the host cell is visible in DIC in the merged image. (**A**) Percentage of co-localization of *E. ictaluri* ECV with the autophagy marker LC3. Data are represented as the mean and standard error of the mean for three independent experiments. Asterisks indicate significant difference between WT and 65ST at different time points. (*** = *p* ≤ 0.01) (**B**) Graphical representation of LC3/-I/LC3-II expression in HKDM infected with either WT *E. ictaluri* or the T3SS mutant 65ST based on the band intensities in the Western Blot (**C**). Asterisks indicate significant difference between treatments following one-way analysis of variance and Bonferroni post-test. (* = *p* ≤ 0.1, ** = *p* ≤ 0.01, *** = *p* ≤ 0.001).

Early after invasion, for both WT and 65ST *E. ictaluri*, the autophagosome marker LC3 was recruited to the ECV of both strains, with 20% of WT and 30% of 65ST sequestered in LC3 positive autophagosomes at 30 min post-infection (Figure 3A). The percentage of WT bacteria located in autophagosomes subsequently decreased to less than 10% at 5 h PI. In contrast, 30% of the ECV in cells infected with 65ST were LC3 positive, which increased significantly to around 60% at 1, 3, and 5 h post-infection (Figure 3A). Immunofluorescent data were confirmed by Western blotting (Figure 3B,C), which also demonstrated significantly greater induction of the membrane-localized, lapidated form, LC3-II, in HKDM infected with 65ST than in non-infected HKDM or macrophages infected with WT (Figure 3B,C). This indicates that lipidation of LC3 to produce the autophagy indicator protein LC3-II occurred in 65ST-infected HKDM but not in WT-infected cells.

### 3.2. Caspase Activation during E. ictaluri Infection of HKDM

**Figure 4:**
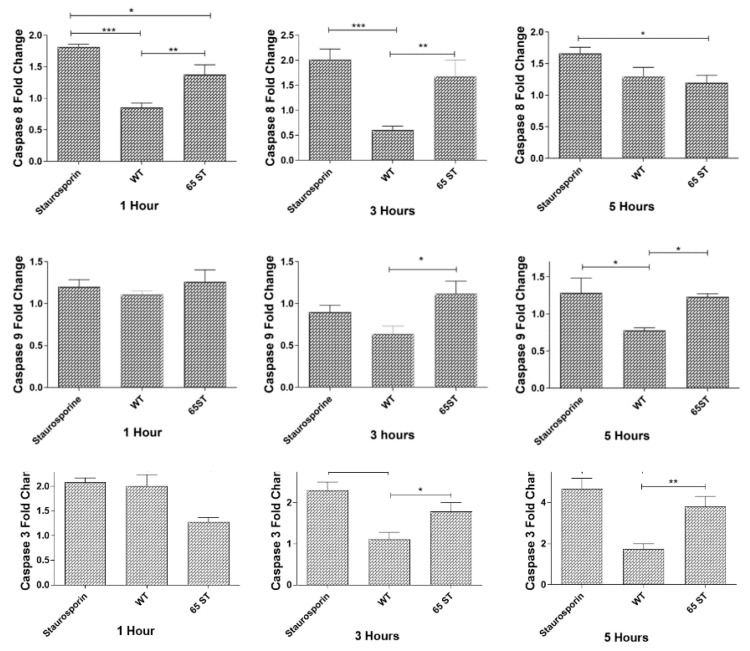
Caspase activities in HKDM cells infected with E. ictaluri WT and T3SS mutant 65ST. Staurosporine treated HKDM were used as a positive control. Cultures were harvested at 1, 3 and 5 h post-infection. Values are expressed as means ± standard deviation of 4 different observations. Asterisks indicate significant differences following one-way analysis of variance with Tukey’s post-test to compare the mean of each treatment with every other treatment. Asterisks indicate significant difference between treatments. (* = *p* ≤ 0.1, ** = *p* ≤ 0.01, *** = *p* ≤ 0.001).

Activity of the initiator caspases, caspase-8 and caspase-9, as measured in fold change from uninfected cells, showed that caspase-8 activity in the T3SS mutant 65ST-infected cells increased at 1 and 3 h post-infection, while caspase-8 activity declined and was significantly lower in WT-infected cells. There were no significant differences in caspase-8 activity after 5 h post-infection. For caspase-9, activity in WT-infected cells was significantly lower than in 65ST-infected cells after 3 and 5 h post-infection (Figure 4). As seen in Figure 4, caspase-3/7 activity increased significantly in the WT-infected HKDM compared to 65ST-infected cells after 1 h post-infection but then declined to a significantly lower level than the 65ST-infected cells at 3 and 5 h post-infection.

**Figure 5:**
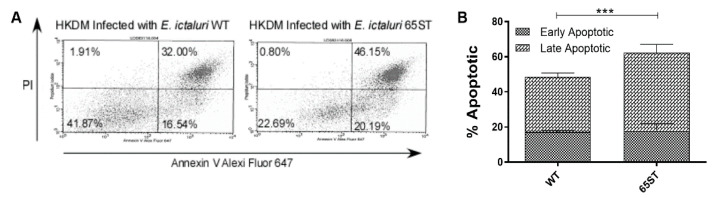
Assessment of HKDM by flow cytometry after annexin V/propidium iodide labeling. (**A**) Results are presented for a representative experiment of three replicated experiments as the percentage of cells that were viable (AnnV+ PI+) in the upper right quadrant. (**B**) Graph of the mean percentages of early and late apoptotic cells for the three experiments shows that the T3SS mutant 65ST was significant greater than the WT. (*** = *p* ≤ 0.001).

The relationship between the T3SS and macrophage apoptosis was further investigated by assessing the surface expression of phosphatidylserine using Alexa Fluor 647-labeled Annexin V (AnnV) in conjunction with propidium iodide (PI) for flow cytometry analysis after 5 h post-infection. As shown in Figure 5A, 41.87% of WT *E. ictalurid*-infected HKDM cells were viable (AnnV-/PI-), significantly more than the 22.69% for 65ST-infected cells. In addition, 16.54% of WT-infected cells were early apoptotic (AnnV+/PI-) and 32.00% were late apoptotic (Ann+/PI+), a total of 48.54% positive for apoptosis. In contrast, infections with 65ST resulted in a significantly greater increase in the level of late apoptotic cells at 46.15% and early apoptotic cells at 20.19% of total cells, a total of 66.34% positive for apoptosis. These results demonstrate that apoptosis is significantly suppressed by the WT strain (Figure 5B).

### 3.3. Cellular Damage in Macrophages Caused by Invasion of E. ictaluri

**Figure 6:**
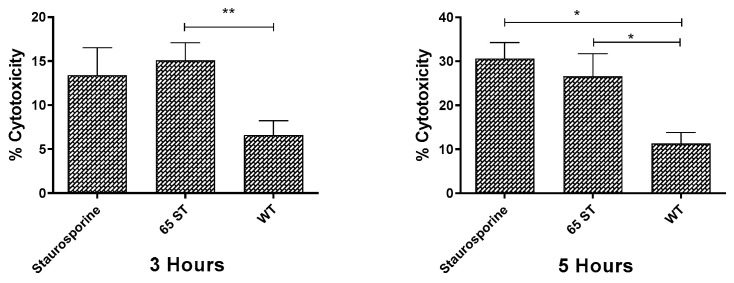
Release of LDH by HKDM infected with WT *E. ictaluri* and the 65ST T3SS mutant for 3 and 5 h post-infection level of cytotoxicity was significantly lower for the WT than for the staurosporine treated cells or the 65ST infected cells at both 3 and 5 h post-infection. (* = *p* ≤ 0.1, ** = *p* ≤ 0.01).

As seen in Figure 6, at 3 h post-infection, only 6.6 ± 1.6% cytotoxicity was detected in HKDM infected with WT and this percentage increased to 11.31 ± 2.5% at 5 h post-infection. In contrast, the cytotoxicity of cells infected with the T3SS mutant 65ST was significantly greater, at 15.08 ± 2.01% at 3 h post-infection and 26.57 ± 5.15% at 5 h post-infection. Cytotoxicity for HKDM treated with staurosporine was not significantly different from HKDM infected with 65ST. 

## 4. Discussion

As previously indicated, *E. ictaluri* can survive and replicate in channel catfish HKDM [1,2,6]. Early survival and replication in the ECV is dependent on bacterially encoded virulence factors, including an acid activated urease [1] and a Salmonella-pathogenicity-island-2 class of T3SS [11,12]. Subsequent development of infection is dependent on the action of bacterial effector proteins that are translocated from within the ECV through the bacterial envelope and the ECV membrane into the host cell cytoplasm by the T3SS [3]. Translocated T3SS effector proteins are known to interact with specific host cell target proteins and subvert host defense mechanisms to the pathogens’ benefit by manipulating host cell processes [13,14,15,16,17]. Based on the presence of the early endosomal markers Rab5 and EEA1, and the late endosomal marker Rab7 in the membrane of ECV, along with the lack of significant levels of Lamp1, the *E. ictaluri* T3SS effectors interrupted maturation of the ECV after early and late endosomal fusion. The presence of the lysosomal marker Lamp1 on 65ST and the absence on the WT ECV indicates that T3SS activity also prevented fusion of the WT ECV with lysosomes.

Replication and vacuolar growth within a host cell by intracellular bacteria requires a source of nutrients for microbial growth, cell division, and expansion of microbial vacuoles. The significantly lower levels of the ER marker calnexin in the WT ECV compared to the ECV of the T3SS mutant 65ST, combined with the significantly greater levels of the Golgi marker giantin in the WT ECV, suggests that membrane material for the growth of the WT ECV was provided primarily by the Golgi rather than the ER and that this change is a product of T3SS effector activity. This change would also provide nutrients from the Golgi vesicles for bacterial growth [18]. The similarity in percentage of both 65ST and WT ECV co-localizing with calnexin at 30 min suggests that both strains picked it up during initial uptake in the manner of ER-mediated phagocytosis, as described by Gagnon et al. [19].

The role of autophagy in a bacterial infection ranges between a host-protective immune-related induction and a host-damaging induction of metabolic changes that render a diseased condition on the host. Traditional autophagy is a degradation process that targets bacteria to a double-membrane vacuole that fuses with lysosomes to form autolysosomes that degrade the offending bacterium. Pathogenic bacteria, however, have developed factors that enable them to avoid autophagy and even exploit it to their benefit [20,21]. Based on the results here, showing limited recruitment of the autophagy marker LC3 to the WT ECV and limited lipidation of LC3-I to LC3-II, *E. ictaluri* has established a mechanism to repress the autophagy pathway and avoid degradation. A significant increase in co-localization of LC3 to vacuoles containing the T3SS mutant 65ST and the inability of 65ST to survive and replicate in HKDM suggests that one or more of the T3SS effectors mediates the *E. ictaluri* ability to subvert LC3 recruitment to the ECV, prevent phosphorylation of LC3-I to LC3-II, and prevent autophagosomal development.

Caspases are cysteine-aspartic acid proteases that play an important role in the initiation and activation of programmed cell death through apoptosis. Caspase-9 triggers intrinsic, or mitochondrial, signaling pathways of apoptosis, while caspase-8 triggers extrinsic or cell surface receptor pathways [22]. Initiation of either of these pathways leads to activation of the executioner caspases-3, -6, and -7, which activates substrates that mediate the changes that characterize apoptotic cells, including cell shrinkage and blebbing, as well as genomic DNA and nuclear fragmentation.

The highly significant and early inhibition of extrinsic initiator caspase-8 at 1 and 3 h PI in the WT-infected cells compared to the lack of inhibition by the T3SS mutant 65ST indicates that the *E. ictaluri* T3SS acts to repress caspase-8 activity that would be induced by attachment of *E. ictaluri* to macrophage surface receptors. This would prevent initiation of apoptosis by the WT and would prevent the subsequent release of LDH in the cytotoxicity assay. At the same time, the intrinsic initiator caspase-9 is essentially unchanged, with weak and late inhibition at 3 and 5 h PI for WT-infected cells. The repression of autophagy is also indicated in the flow cytometry data, where the WT has significantly fewer apoptotic cells than 65ST. Interestingly, executioner caspase-3 is significantly increased at 1 h PI for WT compared to 65ST but then is significantly reduced compared to 65ST at 3 and 5 h. The initial increase and subsequent decrease in caspase-3 in WT could be a result of ubiquitination by the ubiquitin ligase domain of one of the leucine rich repeat (LRR) effectors and subsequent degradation via the proteasome and lysosome.

Given the description of the early trafficking of the ECV and the subsequent lack of autophagy and apoptosis activity of the WT, the model of infection described in the introduction can be expanded. Although the pH of the ECV is downregulated by the vacuolar ATPases of the host cell and then neutralized by ammonia produced by the *E. ictaluri* acid-activated urease, maintenance of the neutral pH conducive to *E. ictaluri* growth and replication is assured by the prevention of phagosomal/lysosomal fusion. The development of the ECV is further enriched by the fusion of Golgi into the vacuolar membrane to provide material for the growing vacuole, as well as Golgi-contained nutrients to support bacterial growth. Finally, suppression of programmed cell death ensures that the infected host cell remains intact.
